# Perceptions of Care Quality during an Acute Hospital Stay for Persons with Dementia and Family/Carers

**DOI:** 10.3390/healthcare9091176

**Published:** 2021-09-07

**Authors:** Lynette Chenoweth, Janet Cook, Anna Williams

**Affiliations:** 1 Centre for Healthy Brain Ageing (CHeBA), University of New South Wales (UNSW), Sydney, NSW 2052, Australia; 2 School of Nursing, University of Notre Dame Australia, Sydney, NSW 2010, Australia; janet.cook@outlook.com (J.C.); anna.williams@nd.edu.au (A.W.)

**Keywords:** dementia, acute hospital, quality, person-centred care, qualitative research

## Abstract

Objectives: to report on acute hospital care experiences for persons with dementia and family/carers in a pilot study (PiP) of person-centred care compared with usual care. Methods: participants were recruited from one acute aged care ward and one mixed medical/surgical ward. One-on-one interviews occurred soon after discharge using a semi-structured interview guide framed by person-centred principles whereby the person is: V—valued; I—treated as an individual; P—perceived as having a unique identity; and S—supported socially and psychologically. Data were analysed deductively with reference to these a priori principles. Results: 11 consented persons with dementia and 36 family/carers participated. A total of eight core VIPS concepts were derived from the data. While many occasions of person-centred care occurred, there was variability in staff expertise, interest and aptitude for dementia care work. Neglect of person-centred principles more frequently occurred for the usual care group, where staff failed to place the person and their family/carer at the centre of service. Conclusions: person-centred services for persons with dementia requires that hospital executive equip staff with the relevant knowledge, skills and support to adhere to person-centred care guidelines. Hospitals must address workplace cultures and procedures that favour organisational systems over person-centred services.

## 1. Introduction

Persons living with dementia need extra care and supervision to stay safe and well during a hospital stay [1,2,3]. Hospitalisation can result in increased cognitive, physical and functional deterioration, prolonged stay and high risk of readmission for these persons [4,5]. Iatrogenic harm risks include falls, sepsis, pressure ulcers, fractures and delirium, and a five-fold increase in mortality rates [6,7]. Unfamiliar surroundings, unknown staff, frequent staff changes and interruptions in daily routines can cause delirium and increased behavioural and psychological symptoms of dementia [8], which further complicate the person’s treatment and care [9]. These risks demand the readiness of hospitals to ensure a dementia-friendly environment and dementia-specific delivery of care [10].

Person-centred care (PCC) is an evidence-based framework for supporting the holistic healthcare needs of people with dementia [11]. PCC differs from the more usual treatment-focused hospital care by paying attention to the person’s psychosocial needs and personhood (self-hood) [12,13]. The Social-Psychological Theory of Personhood in Dementia is the basis for the PCC approach [12]. The theory proposes that people living with dementia exist in a social, relational context and that positive and enriching interpersonal relationships can prevent the disabling effects of dementia and promote well-being. Achievement of well-being is a core aim of all treatment and care for people with dementia in the PCC approach. Since the person’s experiences are impacted by the social-psychological milieu of the acute care setting, the theory proposes that this milieu can have a significant effect on the person’s health, well-being and recovery from illness. PCC aims to reduce the potentially negative influence of the healthcare environment [12]. When armed with PCC knowledge and skills, staff are more likely to understand how dementia can impact the person’s behaviour, for example, which often occurs as a response to the healthcare environment [14] and know how to accommodate changed behaviour in routine care [15].

PCC education and training helps hospital staff to gain confidence [16] and find greater satisfaction in caring for people with dementia [17], assists staff to develop therapeutic communication skills [18], support personhood when providing care and establish dementia-friendly environments [19]. Family/carers report that when educated in PCC, staff encourage and enable them to be involved in treatment and care decisions, which leads to better outcomes for persons with dementia [20].

Despite the widespread endorsement of PCC approaches in clinical guidelines for the care of the person with dementia [2,3,21], the adoption of PCC by hospital staff remains sporadic [22]. Barriers to adoption include professional practices and hospital culture [23], time pressures [24] and power relationships between the health professions [25]. The surgical setting is a particularly difficult setting to institute PCC because of high patient turnover and standardised treatment, recovery and discharge protocols [26]. 

Considering the reported challenges to delivering PCC in the acute hospital setting, the authors conducted a pilot study of the Kitwood model of PCC [12] in one acute tertiary hospital in Australia (PiP study) [27]. PiP employed a non-randomised, 12-month pre/post/follow-up design, comparing observed care quality and clinical outcomes for persons with dementia when provided with PCC, and usual hospital care (Control). This paper reports on the methods and results of follow-up interviews held with a volunteer sample of PiP study participants with dementia and family/carers who regularly visited them in hospital. The objective was to obtain feedback on their perceptions of care quality during hospitalisation.

## 2. Materials and Methods

### 2.1. Ethical Considerations

The PiP study [27] was approved by the human research ethics committees of the health service (2019-ETH08705v.3) and the university (2019-ETH019097S). All persons with dementia and their family/carers who met the relevant eligibility criteria for the PiP study were invited to participate in follow-up interviews. Convenience sampling occurred using an arms-length approach by the issue of study advertisements, participant information statements and consent forms and through the assistance of senior ward staff. Interested individuals were also provided with a verbal explanation of the study aims and procedures by research personnel and were given 1 to 2 days to consider participation before providing written consent.

### 2.2. Participants

Of 47 persons with dementia enrolled in the PiP study, 11 of them were assessed by researchers LC and JC to have the capacity and gave consent to be interviewed (Control *n* = 8, PCC *n* = 3) (score 19–25 out of 30, Montreal Cognitive Assessment (MoCA©) Version 8 [28]. A total of 36 of 45 PiP family/carer participants (Control *n* = 16, PCC *n* = 20) also agreed to be interviewed and gave written consent.

### 2.3. Data Collection

The semi-structured interview questions developed by the PiP researchers were based on the VIPS [13] principles of a person-centred healthcare system, which reflects the Kitwood model of PCC [12] (Table 1). One open-ended question invited participants to talk about other experiences and/or issues with the hospital stay not previously covered. Researcher LC piloted the semi-structured interview questions face-to-face with one of the PiP participants and their family/carer, who made no recommendations for changes to the interview questions, wording or format. 

Three female PiP researchers with qualitative research expertise (LC, JC, AW) individually conducted the one-on-one interviews between 14 to 20 days post-discharge; face-to-face for participants with dementia in their home (mainly a nursing home) and by telephone with family/carers. Interviews took between 50 and 90 minutes, were audio-recorded with the participant’s written permission, transcribed verbatim by researchers LC, JC and AW within 1–5 days and unique identifier codes were allocated to protect participant identity, e.g., PCW01P01 (PCC group, Ward 1, Patient 1) and UCW02FO3 (Usual Care, Ward 2, Family 3). Field notes were not recorded during or following audio-recorded interviews. Transcription accuracy was checked by reading the transcript while listening to the audio recording, and inadvertent mention of the hospital and any person’s name by interviewees was erased. Audio recordings and transcriptions were stored on a password-protected server accessible only to LC, JC and AW. Of 36 family/carers invited, 4 of them requested to inspect their interview transcriptions; none of them requested amendments, deletions or additions. No person with dementia had sufficient intact memory to review and confirm their interview transcripts, and thus all of them declined the invitation. No follow-up interviews were undertaken owing to the potential burden for participants.

### 2.4. Data Analysis

Participant characteristics were descriptively analysed, and interview responses were analysed deductively with reference to the VIPS principles [13]. The data were managed and organised on Microsoft Word and tabulated. Researcher LC coded all transcribed data using the following process: text-familiarisation, data-categorisation according to the VIPS principles, interpretation of data and allocation of data codes, and identification of core concepts derived from the data codes after several inspections of the data. Quotes that matched more than one code were independently reviewed and allocated by researchers JC and LC alongside the interview questions, and then, were jointly reviewed by LC and JC using an iterative process to identify and agree on core concepts that aligned with the 4 VIPS principles. This review process achieved 94% agreement between independent ratings, by dividing the number of agreed codes and core concepts by the number of agreements and disagreements [29]. Researcher AW reviewed the allocated data codes and core concepts alongside supporting quotes and considered their alignment with the VIPS principles. Data saturation was determined when researchers LC, JC and AW agreed that no new codes could be identified from the data and that all codes fitted the core concepts allocated.

## 3. Results

The characteristics of participants with dementia (*n* = 11) and family/carers (*n* = 36) are presented in Table 2 and Table 3.

### 3.1. Interview Findings

Eight core concepts that reflected the four VIPS principles [13] of a person-centred hospital service emerged from the interview data.

The alignment of the data to these eight core concepts are as follows: 

### 3.2. V (Valuing)

#### 3.2.1. Core Concept—Confidence in Staff’s Expertise in Care and Treatment Provision

Participant confidence in staff’s caregiving expertise often referred to staff personalities, staff education and training, and adherence to care protocols that were more suitable for persons with dementia.

Perceptions of staff expertise in care and treatment were more positive for the PCC group than the Control group. Eleven of the PCC group family/carers and two participants with dementia rated both nurses and allied health staff expertise between 6–7 out of 7. Five Control group family/carers and two participants with dementia rated nursing expertise between 6–7 out of 7, and allied health staff received ratings of between 0 and 5 out of 7.

PCC group participants from both Ward 1 (acute aged care) and Ward 2 (medical/surgical) considered that the nurses provided a good standard of care and found “the whole experience was very positive” (W1F34), staff were “very helpful, very experienced” (W1F27), “amazing, fantastic job” (W2F09) and “nursing care was impeccable” (W2F08). Family/carers were particularly impressed with the way staff responded to persons with dementia since, “they understand about him” (W1F32), as were the participants with dementia, “yes, very good, they cared for me” (W1P01), and “always good” (W1P29).

By contrast, control group family/carers rated Ward 2 (medical/surgical) allied health staff as having low expertise (0–4 out of 7), mainly because of patient neglect, with responses such as “I can’t attend to him at the moment” (W1F13), or “I have other patients and I am very busy” (W2F02), being common. Participants with dementia lamented “they should pay much more attention towards the patients’ (needs)” (W1P14) and considered that allied health staff expertise to be “very ordinary” (W2P01). As well, Control group participants gave low ratings for nursing staff expertise because requests for information on treatment plans “were never responded to by any staff member*”* (W1F10) and “staff often said ‘I don’t know, but I will find out’, but no advice was forthcoming” (W2P02).

#### 3.2.2. Core Concept—Safe, Secure and Comforting Care and Treatment Regimens

Participant views about the provision of safe and secure hospital systems referred to having specialist dementia protocols in place, and flexibility in care practices for the most unwell and dependent persons with dementia.

PCC group family/carers considered that the person with dementia felt safe, and was respected and valued, since staff were “caring and kind, well cared for and he looks happy” (W1F24), “very positive towards him, amazing” (W2F09), and “their professionalism, their attitude was admirable” (W1F34). They were particularly happy with the nurses’ positive attitudes towards persons with dementia, where “they tried to accommodate, moving him to a window whenever they could, so that was really good and really one of the most important things for his neurological problems” (W1F32). 

Other positive experiences included staff being “quite sensitive to his wishes” (W1P27), “writing up on the board about certain likes and dislikes of this that would help him” (W1F32) and “offer to walk and feed him, they were treating him as a person rather than a bed number” (W1F13). These perceptions were similar for participants with dementia, “they did everything to help me” (W1P01). 

A few of the control family/carers agreed that the staff “made time to answer questions like ‘What do you use this for?’, ‘How do you use it?’. They were terrific” (W2F08). For the remainder “there were definitely things that stand out to me that were not great, like some of the patients who couldn’t move themselves, would be like calling out, and the nurses would be like-Oh, I’m busy, oh, I’m training” (W2F06). Similarly, participants with dementia reported that staff “disregard, don’t care, slam the door or wake people when they are asleep. I think they should not wake people who are fast asleep and shake them to have something to drink” (W1P14). A common safety concern was when: “some of the elder people who were in mum’s room didn’t get that much care and they were left to wait for quite some time, and ‘Well you’ll just have to wait. We’ve got other people to attend to’, and she was calling and calling and calling and nobody came, and patients who couldn’t cut their food and the food was just left there” (W1F15).

### 3.3. I (Individualised)

#### 3.3.1. Core Concept—Staff Interest in and Attention to the Person’s Treatment Preferences and Unique Care Needs

Participants felt respected and valued in their interactions with the staff when time was made to ask questions about the person’s routines, abilities and requirements, to answer the person’s and family/carer’s questions and to respond empathetically to any concerns.

For the PCC group family/carers, staff attention to the person’s unique care needs and treatment requirements was “really well organized” (W1F36) and “really positive” (W1F20). Staff reportedly consulted and involved family/carers in care, treatment planning, decision-making by seeking information on “his background, what he was up to and what the expectations were in terms of post hospitalisation, care management plan and a good path in terms of a good outcome for him” (W2F10). The nurses were frequently “very open to my input, I was very much involved, they were very accepting, and I think they actually welcomed it” (W1F32). Detailed background information was also obtained from the person with dementia to plan and monitor their care and treatment outcomes: “They also asked her personally about these things and what she wanted, about her shower etc. My grandmother told them all that” (W1F30).

Family/carers were also consulted on medication requirements, such as “what he was on, anti-seizure medication and the electrolytes to balance out his diet, to supplement his diet” (W1F34), and often in relation to safety issues, such as “why she was taking so much antipsychotic medication” (W2F10).

For some Control group family/carers “staff consulted on a range of factors” (W1F12) and sought advice about “expectations and limitations” (W1F16), with nurses *‘*always asking, responding” (W1F12). Nevertheless, most family/carers reported that “I don’t think anybody asked me questions on that level” (W1F17), and ‘we weren’t quite in the loop, a stranger as far as the nurses were concerned” (W1F10).

Staff routines often took precedence over patient care preferences for Control group participants, as one explained, “you’re in a deep sleep and the next thing you’ve got this cold strap around your arm where they’re taking your blood pressure, there’s no argument about it, they just do it.” (W1P27). For another person with dementia, “they ask (about preferred routines), then when it comes it is an entirely different thing” (W2P01). Others complained that “I was told the way they do things here. You have to fit into their routines” (W1P14), and “I was just obliged to do what they wanted me to do.” (W2P02). 

Few family/carers were invited to *“*have a good interactive discussion” (W1F15) on medications and were “given advice on administering*”* medicines (W1F12). Most commonly family/carer requests to be involved in medication discussions saw nurses “ignoring advice” (W1F13), and “not asking me, they just changed one of the medications” (W1F26). One family/carer was accused of “bullying” (W1F13) staff when attempting to discuss medication dose, and another’s advice on the best way to administer a medicine was ignored: “They put Pentasa granules in water and say, ‘Drink it’ and of course he won’t. It’s not going to dissolve, so I said ‘put it in custard, put it in yoghurt’. They just ignored me” (W1F21).

Control group staff disinterest in consulting with the person and their family/carer on medication requirements left them feeling “intimidated” (W2P06, W1P18), resulting in deleterious outcomes for the person: “I brought in a list of all his medicines, with their names and doses and administration times to be taken and gave it to them. They did not follow the list and gave him double the dose of one of his Parkinson’s medicines which was disastrous-he became delirious” (W2F02). 

#### 3.3.2. Core Concept—Perceived Preservation of the Person’s Self-Esteem, Dignity and Identity

Participants considered that staff showed genuine interest in supporting the person’s self-esteem, dignity and identity when they acknowledged the person’s vulnerability and sought to prevent further deterioration in their health and well-being. 

Most of the PCC group family/carers considered that staff attempted to preserve the self-esteem, dignity and identity of persons with dementia, since they “were interested in him, talking to him and asking him questions” (W1F24). Nurses often asked family/carers “to write up on the board about certain likes and dislikes that would help him” (W1F32). They were pleased when the person “is heading in the right direction*”* (W2F10), and where staff were “monitoring and dealing on a one-on-one basis and determining what was needed” (W1F35).

By contrast, for many Control group family/carers, the staff neglect of aspects of personal care impacted the person’s self-esteem and dignity, such as when “they didn’t’ bother washing her hair, they thought it was a trivial thing” (W2F09). While a few family/carers acknowledged that “some staff were sympathetic to her feelings” (W1F15), most of them were dismayed with staff’s failure to support the person’s self-esteem and dignity: “He had delirium and hallucinations and became agitated and aggressive, which the staff seemed to blame on him. They restrained him physically because he needed to get to the toilet, but they didn’t come to help him. They blamed him for what they did to him” (W2F02).

A traumatic experience was reported when a male nurse “manhandled my grandmother quite aggressively, pulled down her pants, shoved her around, she was quite exposed” (W2F06). There was considerable family/carer distress when told by staff ‘We’re not going to do anything. The sooner you agree (to nursing home placement) the sooner we can sort it out” (W1F13). Both nurses and allied health staff were often “inattentive and unresponsive and too busy to respond” (W1P27) and “very blunt, dismissive” (W1F13). Experiences such as these caused many family/carers to reflect “there could have been a little bit more empathy” (W1F08), and “only a few nurses and physios actually cared about him” (W2F02).

With tears, two of the Control group participants with dementia recalled being “treated like an object” (W2P02) and “when I cry and beg, they do nothing” (W1P14). Others were similarly traumatised when their self-esteem and dignity were disregarded by staff who were “regimental sergeant majors” (W1P27), exclaiming “I just hope I never have to go to hospital again” (W2P01) and “It’s the system. The hospital is terrible” (W1P18).

### 3.4. P (Person’s Perspective) 

#### 3.4.1. Core Concept—Acknowledgement of the Person’s Fears, Issues and Concerns

Participants considered that quality hospital systems acknowledged and gave attention to addressing their issues and concerns with service standards, care requirements and restrictive practices.

PCC group participants with dementia and family/carers were frequently given opportunities to be partners in decision-making, in which “there was a whole session, a whole panel with all different staff members” (W1P27). They spoke about how nurses were “monitoring (his emotional state), dealing with him on a one-on-one basis, managing his care, what he needed and how” (W2F10), and adjusting care requirements by “facilitating and then adding or taking away whatever he was happy with, or not happy with” (W1F34). Staff also acknowledged the family/carer’s concerns about the person’s well-being, “saying ‘This is what he has been doing. Is there anything we can do for you?’. Nothing was too much trouble” (W1F13). There were occasions when nurses were sensitive and responsive to the person’s fears and concerns: “there were times when she got very scared at two in the morning and they did let me come in then, and I appreciated that” (W2F06). 

Nevertheless, staff willingness to engage with the person and/or their family/carer “depends on who it was. Some always had a nice smile, others it was like they weren’t happy to see people” (W1F19). A lack of staff sensitivity to participant issues and concerns occurred when “the team did not get to the guts of anything” (W1F15), and staff showed “disinterest, not asking ‘Do you have any problems? Is there anything you’d like to discuss with me?’” (W1F21). Some experienced ostracism for raising concerns about the person’s care “It was like, raised eyebrows and walk in the opposite direction. ‘She’s back!’ They’d see me at the door and walk away” (W1F13), while others’ requests were ignored in situations where “he can’t (choose) and doesn’t understand” (W1F21).

#### 3.4.2. Core Concept—Supporting the Person’s Memories, Strengths and Health Aspirations

Participants considered it important that staff seek to understand the person’s normal routines, abilities and habits to inform the care approach and to encourage and assist the person to achieve their aspirations in the recovery process.

PCC group family/carers frequently recounted ways in which the staff aimed to support the person’s memories and strengths by asking: “questions on his background, what he could do, what he was up to and what our expectations were in terms of post hospitalisation, and (responding with) his care management plan, what they were doing, and what they thought was a good path for him to be able to do for himself” (W1F36). 

Some reported that the staff “asked her (person with dementia) personally, like how she lives in her unit and stuff like that about her mobility and if it was easy to move around and how she has a shower” (W1F30) when seeking to meet the individual’s preferred care goals, which made “my grandmother happy with the care” (W1F30). Participants with dementia considered that the nurses “did try their best” to support their abilities and health aspirations (W1P27). A few Control group participants with dementia found staff to be “interested in finding out about me and my needs” (W2P02) and were “responsive to questions” (W2P01).

Attempts by Control group family/carers to discuss the person’s unique care needs with nurses, however, were frequently unsuccessful, “no, they didn’t, no one seemed to care at all” (W2F06), “not at all, I don’t think they were interested” (W1F15), and nurses did “not respect that I know her, decisions are never discussed” (W2F06). Request for updates on the person’s progress was frequently met with “why are you asking me? I can’t answer any of this!” (W2F07).

A lack of staff interest in supporting the person’s abilities by “putting that nappy on her so she wouldn’t have to call to go to the toilet. They say that haven’t got time to come for that” (W2F09) negatively impacted their independence and dignity. Similarly, participants with dementia reported that staff “refused to help, not interested in those things” (W2P02), and “some ask nothing, they just do what they are supposed to do” (W1P14). Another reflected, “I would have expected that the rehab team would have had an interest in knowing what I can do and what assistance I need to maintain function. But no, nothing” (W2P04).

### 3.5. S (Social and Psychological)

#### 3.5.1. Core Concept—Friendliness of Staff and Attempts for a Therapeutic Relationship

Staff were expected to place value on regular and clear communication with the person and their family/carer about care, treatment and progress, as a way of developing a trustworthy relationship.

PCC group family/carers considered that nurses and allied health staff attempted to build trusting relationships with them and the person with dementia, by “keeping me abreast of it, informed me what was taking place and if I asked questions and they’d give an answer and (discussed) options for him after he got out of hospital” (W1F26). They reported having “had good chats with all of them, they were great” (W1F24), and the “nurses made me feel very welcome” (W1F21). Any distress and uncertainty experienced by persons with dementia and family/carers were “regarded empathetically” by staff (W1F30, W2F10), and the person’s “aspirations for recovery” (W1F32) were supported.

The staffs’ willingness to educate and support family/carers in preparing the person for discharge was “excellent, absolutely terrific” (W2F08), where the “nurses treated me as an equal carer” (W1F20). For one participant with dementia “it (was like being) home again…the pleasure of it all when everyone is on the same page” (W1P27). Some of the Control group nurses also attempted to form trusting relationships with family/carers, being “kind and helpful” (W1F06), “friendly and professional, enjoyed the chats” (W1F12) and “familiarity was comfortable” (W1F16).

Nevertheless, Control group family/carers attempts to engage with staff often “depended on who you spoke to” (W1F08), and staff were often “grumpy … a bit off’’ (W1F08) and found it a “struggle” (W1F27) to obtain “information on progress” (W1F06). Some remarked that there was “too much turnover with nursing staff to build any relationship” (W1F19) and that staff “did not have patience” (W1F15), were “remote and uncaring” (W1F18) and “patronizing” (W1F15). It upset family/carers to recall “the way they spoke to me, almost as if I don’t want to have to talk to you because you are so difficult” (W1F13), and when “certain people display arrogance if you try to give your viewpoint” (W1F16).

Many family/carers found that neither the nursing nor allied health staff accepted their wish to continue the caring role, with offers of assistance being dismissed: “She said ‘You don’t need to be here, we’ll take him up and send him off’. I said ‘I do need to be there, he’ll be confused, he doesn’t know where he’s going. You can’t do that without me being there.’ And she said ‘No, no we’ll just pack it all up” (W1F24).

#### 3.5.2. Core Concept—Responsive Staff and Treatment and Management Regimens

Where staff were seen to place value on the therapeutic nature of their service in line with the person-centred approach to dementia care, participants considered that the person with dementia and their family/carer were treated as individuals and with respect. 

Most of the PCC and a few of Control group family/carers found staff to be responsive to the person’s issues and needs: “They are very dedicated, there’s a lot of compassion, caring, understanding, they have gone through a lot with him and they never got angry, never got frustrated, never had a bad word” (W1F17).

Responsive staff frequently “said hello and smiled and responded to whatever I ask” (W1FO7) and “always ask you if you have any questions, any concerns, always willing to help and if they don’t have the answers they’ll say ‘OK we’ll get back to you’ and see a doctor for you” (W1F12). Other family/carers were pleased when they “made suggestions that it would be better if you do it this way, and most of them would then stop, change and try” (W1F13), and when staff “helped (the person), being prompt in their response and smiling” (W1FO7). Some participants with dementia, too, found staff to be “responsive” (W2P01, W1P27), and “interested in finding out about me and my needs” (W2P02).

It was mainly the Control group family/carers who found nurses and allied health staff to be neglectful: “There is a bit of an attitude of ‘I’m going to sit in the office and I’ve got all this work to do’, as opposed to just going out there and caring about the people that are actually in the hospital” (W2F06).

On occasions “staff were defensive when asked for additional help. I did most of his care, yet they rarely consulted/spoke with me about his care” (W2F02). Lack of staff’s responsiveness caused one to suggest that staff could ”be more proactive instead of him being just another patient. They didn’t explore any options in how to care for someone with dementia in pain” (W1F27). For another, the poorly organised discharge procedure “really upset me. I said ‘Look, he’s not a parcel that you’re sending from one place to another’. There’s got to be some consideration for the state that he’s in” (W1F34). 

Control group participants with dementia reported that “nurses did not talk with me too much” (W1P14), “allied health staff were generally unresponsive” (W2P02), “complaints don’t go anywhere” (W1P02), “their social skills are not very good” (W1P08), and “their looks speak” (W1P14). Participants with dementia reported being “pretty much left alone, they don’t ask me, they tell me what is happening. I go into myself” (W1P01) and being “put in a situation which I was the victim, and ‘You can’t do that, you’re not allowed to do that’” (W1P27).

## 4. Discussion

The VIPS principles [13] provided a useful structure for interpreting the acute hospital experience for persons (participants) with dementia and their family/carers from Kitwood’s person-centred lens [12], in respect of: V—how they are valued; I—how they are treated as individuals; P—how they are perceived as persons with unique identities; and S—how they are supported within the acute hospital service [30]. The eight core concepts uncovered in the data align closely with these four VIPS principles. The findings also concur with the literature that healthcare staff play a vital role in enabling persons with dementia to experience a person-centred hospital service, but there are several barriers to realising this outcome [24].

All participants considered that a quality hospital experience includes staff having the knowledge, aptitude and experience to care for persons with dementia, paying attention to the person’s individual preferences, abilities, needs and routines, and inviting them to be included in treatment, care and decision making [20]. They also expected care and treatment to be focused on the person, rather than on tasks to be undertaken [30]. Participants identified structural limitations in hospital systems, cultures and environments that impeded quality care and treatment [22].

While there remained considerable variability in perceived care quality in respect of staff’s expertise and empathetic responses to the person and their family/carer, the more favourable experiences occurred for the PCC group participants. This finding supports the case for embedding PCC in hospital services for persons with dementia [27,31].

As indicated by the ‘Valuing’ principle, the PCC group nursing and allied health staff placed greater value on dementia care work [16]. Adoption of PCC saw staff providing opportunities for persons with dementia and family/carers to engage in joint treatment and care planning and decision-making [32], and encouragement for family/carers to continue the caring role [33]. Perceptions of staff expertise also included their interest and prompt attention to the person’s needs for comfort and reassurance [24], which resulted in the person’s well-being, safety, dignity and self-esteem [34].

There were notable examples of staff in both PCC and Control groups who respected the VIPS ‘Valuing’ principle, but also reports of staff being uncaring, neglectful and inconvenienced by the person’s requests and human needs. The main difference in the Control group compared with the PCC group, was staff’s inattention to protecting the person’s emotional and physical safety [35]. Indeed, some of the participants were treated as responsible for the symptoms of their condition, such as delirium [30].

In respect of the VIPS ‘Individualised’ principle, it was mainly the PCC group participants who found staff being responsive to the person’s individual requirements and preferences. The more positive experiences indicated that the care was sensitive and responsive to both the person’s physical and psychosocial needs. Where staff were seen to place value on the therapeutic nature of their service, persons with dementia were treated as worthy, unique and as whole persons [24].

When staff failed to acknowledge the person’s individuality in care and treatment decisions, participants expressed distress at having their needs and preferences ignored, with some of them feeling bullied and marginalised [30]. Where staff focused more on task completion than human interaction, the fundamental human needs of the person such as comfort and dignity, were not met. Such neglect of basic human needs is traumatic to persons who depend on staff to meet essential needs [32].

Favourable staff attitudes and respectful approaches towards the person with dementia occurred with positive hospital experiences, again mainly for the PCC group. In alignment with the VIPS ‘Personal perspective’ principle, staff reportedly enabled the person to maintain their abilities, while promoting well-being and self-esteem [22]. Persons with dementia who reported feeling safe, respected and valued, were generally more satisfied that their experiences aligned with their expectations of an effective healthcare system [15].

It was also mainly the PCC group family/carers who staff treated respectfully and regarded them as care partners [36]. Where family/carers were treated as problems to be avoided [20], their requests for case updating and information on procedures were left unanswered. Such experiences signify staff’s ignorance of person-centred principles in service delivery [37].

Participants were unanimous in their expectation that staff would communicate openly and make attempts to form positive relationships with them, as reflected by the VIPS ‘social and psychological’ principle [22]. Expressions of confidence and mutual trust between themselves and staff enabled participants to feel safe when participating in discussions and arriving at mutually satisfactory healthcare decisions [19]. The reverse occurred when staff gave scant regard and attention to the person’s and family/carer’s social and psychological needs, which occurred mainly for the Control group.

### 4.1. Study Strengths and Limitations

While only a small number of PiP participants with dementia were able to participate in the follow-up interviews, their voices and those of their family/carers are given equal weight in critiquing the hospital experience. Judgements made regarding the extent to which hospital services supported the VIPS principles were derived from the participants’ values on what quality hospital services mean for them. Nevertheless, their views are invaluable in making recommendation on reforming hospital policy and practice for persons with dementia.

### 4.2. Future Direction and Research Opportunities

The findings support previous assertions that the person-centredness of hospital care for persons with dementia depends very much on staff knowledge, skill and aptitude for dementia care work, on the hospital systems and work cultures, which shape staff’s approach to this work, and staff’s acknowledgement of the centrality of the person and their family/carer in healthcare decisions. Since some staff seemed constrained by hospital policies and standard procedures, person-centred practice is unlikely to occur unless the hospital executive equips staff and managers with knowledge and skills to overcome these barriers. Future research is required on effective ways to address workplace cultures and procedures that favour organisational systems over person-centred services.

## Figures and Tables

**Table 1:** Patient and family/carer interview guide.

Participant Characteristics	Age, Sex, MoCA Score, Educational Level, Language Spoken at Home, Main Occupation (Current Or Former), Family/Carer Relationship to Person with Dementia, Support Network/s, Family/Carer Visitation Times, Decision-Making Authority.
VIPS Principles	Interview Questions
V (Valuing)	What was your level of confidence with staff’s expertise in care and treatment provision (rated from 0 = no confidence, to 7 = full confidence) What are the reasons for these ratings? What words would you use to describe staff’s attitudes towards you/your family member? What were your experiences of staff’s attention to your/your family member’s safety and comfort?
I (Individualised)	What information did staff obtain from you about your/your family member’s usual activities of living? What information did staff obtain about your/your family member’s likes and dislikes, e.g., food, bathing, routines, comforts? What information did staff obtain about your/your family member’s abilities and needs? How did staff use this information when providing care and treatment?
P (Person’s perspective)	How responsive were the staff to your/your member’s fears, issues and concerns? How responsive were staff in answering questions about your/your family member’s care, treatment and future plans? How responsive were staff to your advice about your/your family member’s care and treatment? Describe your involvement with decision making for yourself/your family member during the hospital stay?
S (Social and psychological)	How friendly were staff to you/your family member? How welcome did staff made you feel? How did the staff respond when you expressed views about your/your family member’s care, treatment and future plans? If you expressed concerns or dissatisfaction about the care or treatment, how did staff respond?
Open-ended question	Are there any other experiences and/or issues that occurred during the hospital stay that you would like to mention?

**Table 2:** Persons with dementia characteristics (*n* = 11).

Characteristics		PCC Group *n*	Control Group *n*	Total N (%)
		3	8	11(100)
MoCA score *	19–25	3	8	11 (100)
Age	60–70	0	1	1 (9)
	71–79	1	1	2 (18)
	80–89	2	6	8 (73)
Sex	Female	1	2	3 (27)
	Male	2	6	8 (73)
Educational level	Primary/Secondary school	0	0	0 (0)
	Higher School Certificate	1	1	2 (18)
	Technical/further education	1	2	3 (27)
	University degree	1	5	6 (55)
Language spoken at home	English	2	6	8 (73)
	Other language	1	2	3 (27)
Previous occupation	Professional/Academic	1	3	4 (36)
	The Arts/Technical	1	3	4 (36)
	Business/Managerial	1	2	3 (27)
Discharge destination	Own/family home	2	2	4 (26)
	Long-term assisted care	1	6	7 (64)
Support systems	Family/friends	2	5	7 (64)
	Community networks	1	3	4 (26)

* MoCA Score: 19–25 (mild-moderate cognitive impairment).

**Table 3:** Family/carer baseline characteristics (*n* = 36).

Characteristics		PCC Group *n* (%)	Control Group *n* (%)	Total N (%)
		20 (56)	16 (44)	36 (100)
Age	18–30	0 (0)	2 (13)	2 (6)
	31–50	4 (20)	3 (20)	7 (19)
	51–70	8 (40)	8 (53)	16 (44)
	≥71	8 (40)	3 (30)	11 (31)
Sex	Female	12 (60)	10 (63)	22 (34)
	Male	8 (40)	6 (37)	14 (64)
Relationship to person with dementia	Spouse/partner	7 (35)	4 (27)	11 (31)
	Son/daughter	9 (35)	8 (35)	17 (47)
	Other family	2 (10)	2 (13)	4 (11)
	Friend	1 (5)	2 (12)	3 (8)
Educational level	Primary/Secondary school	1 (5)	3 (20)	4 (11)
	Higher School Certificate	5 (25)	2 (13)	6 (17)
	Technical/further education	2 (10)	4 (21)	6 (17)
	University degre	12 (60)	8 (53)	20 (56)
Language spoken at home	English	18 (90)	13 (87)	31 (86)
	Other language	2 (10)	3 (19)	5 (14)
Main occupation	Professional/Academic	15 (75)	12 (80)	11 (31)
	The Arts/Technical	8 (40)	7 (44)	15 (42)
	Business/Managerial	2 (10)	3 (19)	5 (14)
	Homemaker/Farming	1 (5)	2 (13)	3 (8)
Support network	Family	15 (75)	12 (75)	27 (75)
	Friends	13 (65)	10 (63)	20 (43)
	Neighbours	6 (30)	3 (19)	9 (25)
	Colleagues	4 (20)	1 (6)	5 (14)
Time of hospital visits	Morning	12 (60)	8 (33)	13 (23)
	Afternoon	13 (65)	6 (38)	19 (53)
	Evening	4 (20)	4 (25)	8 (22)
	All day/night	2 (10)	3 (19)	5 (14)
Decision-maker of person’s hospitalisation	Family/carer	10 (50)	5 (31)	15 (42)
	GP/other doctor	7 (35)	4 (25)	11 (31)
	Nurse/manager	3 (15)	5 (31)	8 (22)
	Ambulance staff	4 (20)	2 (13)	6 (17)

## Data Availability

The study data are not available to the public, as per the written consent clause provided by study participants.
